# Involvement of the Voltage-Gated Calcium Channels L- P/Q- and N-Types in Synapse Elimination During Neuromuscular Junction Development

**DOI:** 10.1007/s12035-022-02818-2

**Published:** 2022-04-27

**Authors:** Neus Garcia, Pablo Hernández, Maria A. Lanuza, Marta Tomàs, Víctor Cilleros-Mañé, Laia Just-Borràs, Maria Duran-Vigara, Aleksandra Polishchuk, Marta Balanyà-Segura, Josep Tomàs

**Affiliations:** Facultat de Medicina i Ciències de la Salut, Unitat d’Histologia i Neurobiologia (UHNEUROB), Universitat Rovira i Virgili, Sant Llorenç 21, 43201 Reus, Spain

**Keywords:** Motor endplate, Postnatal synapse elimination, Axonal competition, Protein kinases, VGCC

## Abstract

During the nervous system development, synapses are initially overproduced. In the neuromuscular junction (NMJ) however, competition between several motor nerve terminals and the synapses they made ends with the maturation of only one axon. The competitive signaling between axons is mediated by the differential activity-dependent release of the neurotransmitter ACh, co-transmitters, and neurotrophic factors. A multiple metabotropic receptor-driven downstream balance between PKA and PKC isoforms modulates the phosphorylation of targets involved in transmitter release and nerve terminal stability. Previously, we observed in the weakest endings on the polyinnervated NMJ that M_1_ mAChR receptors reduce ACh release through the PKC pathway coupled to an excess of Ca^2+^ inflow through P/Q- N- and L-type voltage-gated calcium channels (VGCC). This signaling would contribute to the elimination of this nerve terminal. Here, we investigate the involvement of the P/Q-, N-, and L-subtype channels in transgenic B6.Cg-Tg (Thy1-YFP)16-Jrs/J mice during synapse elimination. Then, the axon number and postsynaptic receptor cluster morphologic maturation were evaluated. The results show that both L- and P/Q-type VGCC (but not the N-type) are equally involved in synapse elimination. Their normal function favors supernumerary axonal loss by jointly enhancing intracellular calcium [Ca^2+^]_i_. The block of these VGCCs or [Ca2+]i  i sequestration results in the same delay of axonal loss as the cPKCβI and nPKCε isoform block or PKA activation. The specific block of the muscle cell’s contraction with μ-conotoxin GIIIB also delays synapse maturation, and thus, a retrograde influence from the postsynaptic site regulating the presynaptic CaV1.3 may contribute to the synapse elimination.

**Supplementary Information:**

The online version contains supplementary material available at 10.1007/s12035-022-02818-2.

## Introduction

During the nervous system development, synapses are overproduced though only consolidate appropriate connections [1–6]. At the neuromuscular junction (NMJ), various motor axons compete to make stable synaptic contacts with the maturation of only one presynaptic axon and the elimination of the others [7–11]. The activity-dependent release of acetylcholine (ACh), adenosine, neurotrophins, and other mediators allows the mutual influence between axons fitted with the corresponding receptors [3, 11–14]. The axonal competitive signaling is mediated by at least presynaptic muscarinic ACh autoreceptors (mAChR; M_1_, M_2_, and M_4_ subtypes), adenosine receptors (AR; A_1_ and A_2A_), and the tropomyosin-related kinase B (TrkB) neurotrophin receptor [15–20]. A_1_, M_1_, and TrkB operate mainly through the protein kinase C (PKC) pathway, whereas A_2A_, M_2_, and M_4_ are coupled to the protein kinase A (PKA) pathway [4, 21–23]. It has been described that PKA activity opposes to NMJ maturation while PKC promotes axonal loss [24].

The motor nerve terminals achieve differences in both transmitter release and expression of related molecules during the process of the developmental retraction of supernumerary axons that could be the cause of the elimination or survival of the nerve terminals. A metabotropic receptor-driven balance between PKA and PKC activities in the competing axon terminals would be relevant in developmental synapse elimination by the phosphorylation of pre- and postsynaptic targets involved in transmitter release and nerve terminal stability such as VGCC. Related with this, we know that in the weakest endings from polyinnervated NMJ (those nerve terminals that evoke the small synaptic potential), M_1_ mAChR receptor subtype reduces release through the PKC pathway coupled to an excess of Ca^2+^ inflow through P/Q-, N-, and L-type VGCC (L and N channels are present in these weak endings) [25, 26]. Moreover, in these weak nerve terminal, P/Q and N channels enhance release through the PKA-associated M_2_ subtype [22, 27]. Therefore, it is tempting to speculate on the relevance of the PKA and PKC phosphorylation of the Ca^2+^ channels to differentially control the neurotransmitter release and its influence in the nerve terminal stability and loss.

Here, we evaluate the strength of the hypothesis of the close relation between serine/threonine kinases and VGCC for developmental synapse elimination. First, we investigate the involvement of the P/Q-, N-, and L-subtypes of the VGCC, and second, we compared the effect of the channels activity modulation with the effect of PKA and PKC modulation. The results show that both L- and P/Q-type but not the N-type VGCC intervene in the postnatal axonal disconnection and synapse maturation. Their block at the half-time period of axonal elimination strongly prolongs both multi-innervation and postsynaptic AChR cluster immaturity. This effect is not different from the block of the classical cPKCβI isoform and from the PKA stimulation though nPKCε block results in a significantly greater delay suggesting some relevance of this calcium-independent isoform, thus encouraging new experiments to explore these links and mechanism. In addition, a retrograde influence from the muscle cell may contribute because the contraction block with μ-conotoxin GIIIB also delays axon loss and synapse maturation. Furthermore, this retrograde influence may regulate the presynaptic CaV1.3 action on the synapse elimination.

## Materials and Methods

### Animals

B6.Cg-Tg (Thy1-YFP) 16Jrs/J (Thy1-YFP-16) transgenic mice and C57BL/6 J (wild-type control) from the Jackson Laboratory were used. Thy1-YFP-16 express high levels of yellow fluorescent protein in motor and sensory neurons, as well as in subsets of central neurons. Axons are strongly and specifically stained by this line. No expression is detectable in nonneural cells. Thy1-YFP-16 mice were used in all experiments, and in some cases, we checked our results with C57BL/6 J mice. No significant differences were found with YFP mice. Animal-involving procedures were approved by the Ethics Committee of Animal Experimentation of the Universitat Rovira i Virgili and Generalitat de Catalunya (reference number 10760). The animals were cared for in accordance with the European Community’s Council Directive of 24 November 1986 (86/609/EEC) for the humane treatment of laboratory animals. Pups of either sex were used in experiments in postnatal day 9 (P9). The date of birth was designated postnatal day 0 (P0). Conception timing and weights at P9 neonatal mices were carefully monitored to reduce the variability in our measurements. Whole *Levator auris longus* (LAL) muscles were used to perform the morphological analysis at postnatal day 9.

### Western Immunoblotting

For immunoblotting, dissected LAL neonatal muscles (P5, P7, and P30; 1/10 w/v) were homogenized with an overhead stirrer (VWR International, Clarksburg, MD) in ice-cold lysis buffer (NaCl 150 mM, Tris–HCl 50 mM (pH 7.4), EDTA 1 mM, NaF 50 mM, PMSF 1 mM, Na_3_VO_4_ 1 mM; NP-40 1%, Triton X-100 0.1%, and protease inhibitor cocktail 1% (Sigma, Saint Louis, MO, USA)). After the extraction of the insoluble material by centrifugation at 4000 g for 5 min, the samples were centrifuged at 15,000 g for 15 min, and the final supernatants were the lysate samples.

Protein concentrations were determined using the DC protein assay (Bio-Rad, CA, USA). Samples (30 μg of protein) were electrophoresed on 8% SDS–polyacrylamide gels [28] and transferred to polyvinylidene difluoride (PVDF) membranes (Amersham-Pharmacia, Upsala, Sweden). The PVDF membranes were blocked in 5% nonfat dry milk in tris-buffered saline (50 mM Tris at pH 7.4, 200 mM NaCl, 0.1% Triton X-100, 0.2% Tween-20). Primary antibodies were incubated at 4 °C overnight (rabbit anti-P/Q-type calcium channel (1:1000; ACC-001, Alomone; Jerusalem, Israel); rabbit anti-α1D L-type calcium channel (CaV1.3, 1:500; ACC-005, Alomone, Jerusalem, Israel); rabbit anti-N-type calcium channel (1:500; ACC-002, Alomone); rabbit anti-Munc18-1 (1:1000; ≠ 13414, Cell Signalling Technology; Massachusetts, USA), and rabbit anti-PKCε (1:1000; ≠ 2683, Cell Signalling Technology; Massachusetts, USA)). Horseradish peroxidase-conjugated secondary antibody from Jackson ImmunoResearch (Philadelphia, PA) was used at a dilution of 1:10.000 for 1 h. Chemiluminescence was revealed with an ECL kit (GE Healthcare Life Sciences, UK) and imagined with the ChemiDoc XRS + Imaging System (Bio-Rad, CA, USA). ImageJ software was used to calculate the optical density of the bands, always from the same immunoblot image. The values were normalized to (a) the background values and (b) the total protein transferred on the PVDF membranes, analyzed with Sypro Ruby protein blot stain (Bio-Rad, CA, USA) [29]. Means between different postnatal days were calculated from the same membrane image. We compare P7 and P30 from P5. Data was taken from densitometry measurements made in at least three separate Western blots for each of the five animals on each postnatal day. For Western blot design, no blinding was performed.

The specificity of anti-α1D L, P/Q, and N VGCC antibodies used in this study has been tested using KO mice by Alomone Labs and validated also by some researchers. The specificity of the anti-CACNA1A (CaV2.1 or P/Q VGCC) antibody was validated by Jung et al. [30] through the immunohistochemical staining of Cav2.1 in the mouse hippocampus, comparing the expression between a control group and a conditional knockout group (Cav2.1 cKO). The Cav2.1 expression was significantly reduced in Cav2.1 cKO. The antibody specificity was also validated by Alomone Labs through Western blot analysis of the rat brain membranes, using CACNA1A/Cav2.1 Blocking Peptide as a negative control. Anti-α1D L VGCC (Cav1.3, CACNA1D) antibody specificity was determined by Shi et al. [31] using a Cav1.3 knockout mouse (Cav1.3^−^/^−^). The immunohistochemical reactivity of Cav1.3 in a mouse eye section of a Cav1.3^−^/^−^ was not detected in comparison to the Cav1.3^+^/^+^ wild type (WT). The antibody specificity was also validated by Alomone Labs through Western blot analysis of the rat brain membranes, using Cav1.3/CACNA1D Blocking Peptide as a negative control. Fossat et al. [32] also determined the specificity of the antibodies against Cav1.3 by probing the spinal cord for Cav1.3 expression following channel knockdown using a peptide nucleic acid–based antisense strategy. The specificity of the anti-CACNA1B (Cav2.2) antibody was determined by Alomone Labs through Western blot analysis of the rat brain membranes. In addition, to ensure primary antibody specificity in Western blotting, we used two different negative controls (see supplementary information from Fig. 2). One of them was without primary antibody where the membranes never revealed staining due to the secondary antibody. Second, preincubation with the specific blocking peptide (ratio between the antibody and the blocking peptide 1:1; examples from Cav1.3/CACNA1D blocking peptide (#BLP-CC005) and CACNA1A/Cav2.1 blocking peptide (#BLP-CC001)) in skeletal muscle tissue (P5 and P30) prevented immunolabeling. The specificity of the Munc18-1 antibody (#13414,) and PKCε (22B10) antibody (#2683) for Western blots had been tested and published in previous works [33, 34]. For example, the incubation with the specific εV_1-2_ peptide for 30 min decreases PKCε and pPKCε levels [33]. As a positive control, brain lysate was used to detect a specific band from α1D-L-type VGCC, P/Q-type VGCC, nPKCε, and Munc 18–1 (see supplementary information from Fig. 2). In addition, the specificity of α1D L-type VGCC antibody was confirmed when we compared the region of the muscle tissue enriched in NMJ with the peripheral regions without synapses. In the LAL muscle, a clearly defined separation between these regions is not easily performed (differing from other muscles such as the sternocleidomastoid or diaphragm muscles with narrow neural central bands). However, we analyzed in some muscles the two portions for the presence of TRITC-α-BTX labeled AChRs clusters to guarantee a correct separation.

### Injection Procedure

To determine the involvement of calcium channels on the synapse elimination process by morphological analysis, subcutaneous injections of appropriate solutions (activators and inhibitors) were administered. The animals received an injection (50 μL) from P5 to P8 over the LAL muscle, in the back of the neck as previously described [4, 35]. The muscles were dissected and processed on day P9.

Activators and inhibitors of VGCC, PKC, and PKA were diluted to the appropriate concentration in phosphate-buffered saline (PBS). We made experiments to discriminate the postsynaptic involvement in the axon loss regulation. Muscle contraction was blocked with µ-conotoxin GIIIB (µ-CgTx-GIIIB, Alomone Labs Ltd, Jerusalem, Israel). This toxin selectively inhibits sarcolemmal voltage-dependent sodium channels (VDSCs) without affecting synaptic ACh release and synaptic events [36]. The working concentration was 1.5 mM, and the same protocol as for the other substances was used. Different control experiments were done to know if the injection procedure and the PBS or DMSO (Sigma-Aldrich, Saint Louis, MO, USA) diluent change the NMJ morphology. PBS injected in the muscles did not reveal differences with the non-injected LALs in either the number of axons per endplate or nAChR cluster morphology. No changes were induced by the injection procedure in the overall morphology of the motor endplate and nerve terminals (*p* > 0.05, Fisher’s test; data not shown. As the final concentration, 0.1% (*v*/*v*) of DMSO has been used in control and drug-treated preparations. In control experiments, the injection of 0.1% of DMSO over the LAL muscle did not affect any of the parameters studied (data not shown). The solutions were administered at a concentration in accordance with the reported biological action of the substances [37, 38].

### Tissue Preparation and Histochemistry

At P9, after a lethal dose of 2% tribromoethanol (Sigma-Aldrich; Saint Louis, MO, USA), the heads from neonatal pubs were removed and fixed for 1.5 h in 4% paraformaldehyde (Sigma-Aldrich; Saint Louis, MO, USA) and rinsed 3 × in PBS. Then, LAL muscles were dissected and post-fixed for 45 min. Next, Thy1-YFP-16 LAL muscles were incubated (1 h at room temperature) with tetramethylrhodamine conjugated α-bungarotoxin (TRITC-α-BTX, PBS containing a 1/1000 dilution of 1 µg/mL; Molecular Probes; Oregon, USA).

C57BL/6 J LAL muscles were processed for immunostaining to detect the presynaptic motor neuron terminals and the postsynaptic nicotinic acetylcholine receptors (nAChRs). Muscles were incubated in 0.1% glycine (Sigma-Aldrich; Saint Louis, MO, USA) for 12 h at 4 °C and then blocked in a solution containing 4% BSA (Sigma-Aldrich; Saint Louis, MO, USA) and 0.5% Triton X-100 (Sigma-Aldrich; Saint Louis, MO, USA) in PBS for 12 h at 4 °C. Primary antibodies against 200-kD neurofilament protein (rabbit antibody, 1:1000; Sigma-Aldrich) were diluted in a solution of 4% BSA diluted in PBS with 0.5% Triton X-100 and incubated at 4 °C overnight. Tissues were rinsed 3 × in PBS and treated with TRITC-α-BTX (Molecular Probes; Oregon, USA) to label postsynaptic nicotinic acetylcholine receptors (nAChRs) for 45 min. The presynaptic motor neuron terminals labeled by anti-neurofilament protein were visualized by the secondary antibody Alexa-fluor 488 donkey anti-rabbit (1/300; Molecular Probes; Oregon, USA). As a control, the antibody specificity was tested by incubation in the absence of primary antibody. No unspecific staining was observed in the three muscles used as negative controls (not shown). Whole muscles were mounted in Mowiol (Calbiochem-Merck; Kenilworth, NJ, USA) with p-phenylenediamine (Sigma-Aldrich; Saint Louis, MO, USA).

**Fig. 1:**
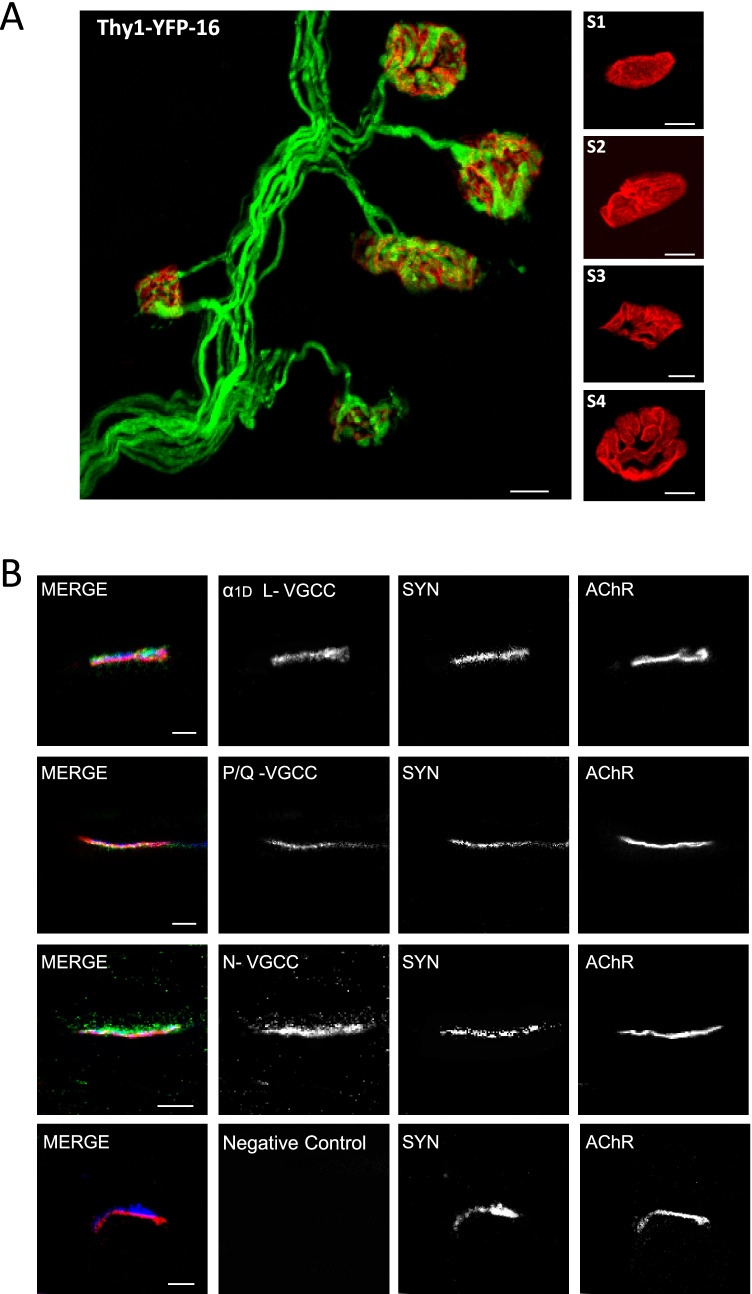
**a** Representative confocal image of a nerve terminal arborization. Singly, dually, and innervated by three or more axons NMJs from YFP muscles and also images of the morphologic maturation (S1, the most inmature, and S4, almost fully differentiated, stages) of the postsynaptic clusters from P9 mice. The bar indicates 10 μm. **b** Confocal immunofluorescence location of α_1D_ L-, N-, and P/Q-type voltage-dependent calcium channels (VDCCs) at the NMJ. Triple labeling of VDCCs (green fluorescence) with syntaxin (blue fluorescence) and nAChR-α-bungarotoxin (red fluorescence) in merge images. Figure shows the presence of α_1D_ L-, N-, and P/Q-type-VDCC (in green) in the nerve terminal of P9 *Levator auris* *longus* (LAL) muscle endplates. The bar indicates 10 μm

VDCCs were detected at P9 neonatal LALs by plastic-embedded semithin sections for high-resolution immunofluorescence analysis [39] and confocal microscopy. We proceeded with the muscles to simultaneously observe α1D L-, N-, and P/Q-type voltage-gated Ca^2+^ channels with nAChR and syntaxin. Muscles were incubated overnight at 4 °C with anti-Ca_V_1.3 (CACNA1D) antibody voltage-dependent L-type calcium channel subunit α_1D_ (1/100; ACC-005, Alomone Labs, Jerusalem, Israel); anti-Ca_V_2.1 (CACNA1A) antibody voltage-dependent P/Q-type calcium channel subunit α_1A_ (1/100; ACC-001, Alomone Labs, Jerusalem, Israel); anti-Ca_V_2.2 (CACNA1B) antibody voltage-dependent N-type calcium channel subunit α_1B_ (1/100; ACC1-002, Alomone Labs, Jerusalem, Israel), and anti-mouse syntaxin (1/1000, S066, Sigma, St Louis, MO, USA). After incubation with primary antibodies, the muscles were incubated for 6 h in a mixture of second antibodies conjugated with Alexa Fluor 488 and Alexa Fluor 647 (Molecular Probes, Oregon, USA). Postsynaptic acetylcholine receptors (nAChRs) were labeled by tetramethylrhodamine alpha-bungarotoxin (1/1000, TRICT-α-BTX, Molecular Probes, Oregon, USA). Then, the muscles were dehydrated with increasing concentrations of ethanol and acetone, and the tissue fragments were embedded in Spurr’s resin in a transverse orientation. Sections 0.5–0.7 μm thick were cut with a Reichert Ultracut E microtome (Leica Microsystems, Bannockburn, IL, USA) and flattened on glass slides by heating on a hotplate. Various different types of negative controls were used to test the specificity of the VDCC antibodies, and at least three muscles were used in each control. In the first control was to omit the primary antibodies. In the second control, the primary antibody was preincubated with the peptides for 2 h prior to use. In the last control, muscles were incubated omitting either one of the two primary antibodies to show a possible cross-linking between the primary antibodies that joined the secondary antibodies. No cross-reaction was detected between antibodies (Fig. 1b). NMJs were viewed with an inverted Nikon TE-2000 microscope (Nikon Japan).

### Morphological Analysis and Confocal Microscopy

The NMJs on LAL muscles were viewed using an inverted Nikon TE-2000 fluorescent microscope (Nikon, Tokyo, Japan) connected to a personal computer running image analysis software (ACT-1, Nikon). The number of axons per endplate was counted and classified into three groups: junctions that were monoinnervated, doubly innervated, or innervated by three or more terminal axons. At the same time, the percentage of immature nAChR clusters was defined as the uniform, density-homogeneous nAChR oval plaques observed at birth, without inhomogeneities in the receptor density or the presence of initial gutters.

High-resolution confocal images were obtained with a 63 × oil objective (1.4 numerical aperture) on a Nikon TE-2000 confocal microscope. Z stacks were obtained at step sizes of 0.5 µm for depths of 20–40 µm, and additional optical sections above and below each junction were collected to ensure that the entire synapse was included.

### Statistical Analysis

Data from Western blot analysis are expressed as means ± standard deviation (SD). Statistical significance was evaluated under a nonparametric Kruskal–Wallis test followed by Dunn’s post hoc test. The criterion for statistical significance was *p < 0.05, **p < 0.01, and ***p < 0.005. Fisher’s test and Bonferroni correction were applied to compare percentages in the morphological analysis. NMJs visible in their entirety were scored, with a minimum of 100 per muscle. In total, 12 muscles were studied for each condition examined. The criterion for statistical significance was* p* < 0.05. The categories were scored, and the counting was performed by a person with no knowledge of the age or treatment of the animals. The data are presented as percentages of NMJ ± SD.* *p* < 0.05, ** *p* < 0.01, and *** *p* < 0.005.

## Drugs

### Calcium Channel Modulators

#### Antagonists, Blockers, or Inhibitors

The stock solutions were Nitrendipine (NT, a L-type channel blocker N144, Sigma-Aldrich) 50-mM; ω-conotoxin-GVIA (ω-CON, N-type channel blocker C9915**,** Calbiochem) 1 mM; and ω-Agatoxin IVA (ω-AGA, a P/Q-type channel blocker, STA-500, Alomone) 100 nM. The working solutions used were NT, 1 µM; ω-CON, 1 µM; and ω-Aga-IVA, 100 nM.

#### Agonist

The stock solutions were 50 mM 1,4-Dihydro-2,6-dimethyl-5-nitro-4-(2-trifluoromethylphenyl)pyridine-3-carboxylic acid methyl ester (Bay-K8644, agonist L-type calcium channel, B-350, Alomone) and 20 mM (2R)-2-[(6-{[(5-methylthiophen-2-yl)methyl]amino}-9-propyl-9H-purin-2-yl)amino]butan-1-ol (GV-58, activator of Ca_V_2.2 and Ca_V_2.1 Ca^2+^ channels; G-140-Alomone). The working solutions used were Bay-K8644, 5 µM, and GV-58, 20 µM.

### Calcium Ion Modulators

The stock solution from BAPTA-AM (1,2-Bis(2-aminophenoxy)ethane-N,N,N',N'-tetraacetic acid tetrakis acetoxymethyl ester) was 10 mM, and the working solution used was 5 µM. BAPTA is a Ca^2+^ chelator with 105-fold greater affinity for Ca^2+^ than for Mg^2+^. BAPTA-AM is a cell-permeable analog of BAPTA that binds calcium only after the acetoxymethyl group is removed by cytoplasmic esterases. It is commonly used at 5–100 μM to evaluate the role of intracellular calcium in cell signaling [40–42].

### Selective PKC Substances

#### Antagonists

The stock solutions were chelerythrine (Che, C-400, Alomone), 10 mM; calphostin C (CaC, C6303, Sigma-Aldrich), 2.5 mM; peptide βIV_5–3_ (βIV_5–3_ Mochly Rosen, Stanford University), 10 mM; and peptide εV_1–2_, (εV_1–2,_ 539522, Calbiochem), 1 mM. The working solutions used were Che (1 µM); CaC (200 nM); βIV_5–3_ (10 µM); and εV_1–2_ (10 µM).

#### Agonists

Bryostatin-1 (BRY, 2283-Tocris; Minneapolis, MN, USA), 10 µM; phorbol 12-myristate 13-acetate (PMA, P1585 Sigma), 10 mM; 12-deoxyphorbol-13-phenylacetate-20-acetate (dPPA, PKCβI selective activator, BML-PE-182–0001 Enzo; Farmingdale, NY, USA), 1 mg/mL; 2-((2-pentylcyclopropyl)methyl) cyclopropaneoctanoic acid (FR236924, PKCε selective activator), 100 mM. The working solutions used were BRY (1 nM); PMA (10 nM); dPPA (0.2 µg/mL); and FR236924 (100 nM).

### Selective PKA Substances

#### Antagonists

The stock solutions were dihydrochloride (H89, 19–141, Millipore-Merck), 5 mM; 8-bromoadenosine-3′,5′-cyclic monophosphorothioate, Rp-isomer sodium salt (Rp8, RI-PKA selective, 129735–00-8, Biolog; California, USA), 5 mM; and adenosine-3′,5′-cyclic monophosphorothioate, Rp-isomer sodium salt (Rp, RII-PKA selective A002S, Biolog), 5 mM. The working solutions used were H89 (5 µM); Rp-8-Br-cAMPS (100 µM); and Rp-cAMPs (100 µM).

#### Agonist

The stock solution was adenosine 3′,5′-cyclic monophosphorothioate,8-bromo-, Sp-isomer, sodium salt (Sp8Br, 116,818, Calbiochem-Merck), 5 mM. The working solution was 10 µM.

Stock solutions were prepared using PBS or DMSO in accordance with the commercial product information. All these solutions are referenced as specific, but possible nonspecific effects of inhibitors and stimulators cannot be discarded.

### Antibodies

Anti-Ca_V_1.3 (CACNA1D) antibody voltage-dependent L-type calcium channel subunit α_1D_ (WB: 1/500; ACC-005, Alomone); anti-Ca_V_2.1 (CACNA1A) antibody voltage-dependent P/Q-type calcium channel subunit α_1A_ (WB: 1/1000; ACC-001, Alomone); anti-Ca_V_2.2 (CACNA1B) antibody voltage-dependent N-type calcium channel subunit α_1B_ (WB: 1/1000; ACC1-002, Alomone); Munc18-1 antibody (WB: 1/1000; #13414, Cell Signalling Technology); PKCε (22B10) antibody (WB: 1/1000; #2683, Cell Signalling Technology).

## Results

### Polyneuronal Innervation in Developing NMJ

Maturation of the NMJ, involving axonal competition and loss of nerve terminals but one, takes place during the first two postnatal weeks. We have selected the period P5–P9 as it corresponds to the *middle* of the axonal loss process. The nerve terminal elimination coincides with the morphological maturation of the postsynaptic component on the NMJ. From the beginning, a uniform nAChR distributed oval plaque (S1) modifies into an increasingly structured pattern of independent primary gutters (S4) [4, 43–45]. Figure 1a shows some representative confocal fluorescence images of singly- and polyinnervated NMJs from Thy1-YFP-16 P9 mice and the morphologic maturation (S1–S4 stages) of the postsynaptic clusters. In addition, Fig. 1b shows the location of α_1D_-L-, N-, and P/Q-type-VDCC at the NMJ. By triple labeling of those proteins (green fluorescence, in merge) with syntaxin (blue fluorescence, in merge) and nAChR-α-BTX (red fluorescence, in merge), we saw that the molecules were present at the neuromuscular contacts, in particular in the nerve terminal.

### VGCC Proteins in Muscle During Development

**Fig. 2:**
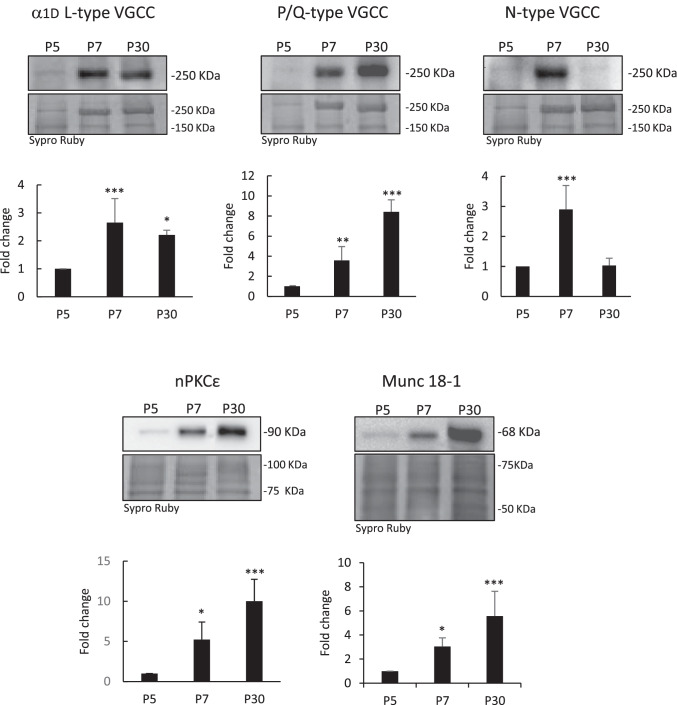
Western blots and histograms of α1D-L-, N-, and P/Q VGCC proteins in the LAL muscle of mice during development (P5-P7-P30). The developmental change in the P/Q VGCC protein level is parallel with the changes observed in other presynaptic molecules (nPKCε isoform and Munc18-1). Data are mean value ± SD, *p < 0.05, **p < 0.01, ***p < 0.005 (*n* = 5; 3 repeats)

We analyzed by Western blotting the changes in α1D L-, N-, and P/Q VGCC protein levels in the LAL muscle of Thy1-YFP-16 mice during development (P5-P7-P30). To control the changes in protein translation during muscle development, our data has been normalized against the average concentration of proteins stained with the highly sensitive Sypro Ruby total protein stain [29]. Figure 2 shows a conspicuous increase of all three channel proteins at P7 that roughly triplicate the value of P5. At P30 stage, the α1D-L-type protein stabilizes at the levels reached at P7. WB from the central region of muscle fibers (containing the NMJ) compared to peripheral regions (almost lacking NMJs) strongly indicates that the α1D-L-VGCC band in the WB represents α1D-L-VGCC in the nerve terminals. The P/Q-type channel protein experiences an important rise at P30 in concordance with their specific involvement in transmitter release in the adult NMJ whereas the N-type protein level falls back to the low value observed at P5The developmental change in the P/Q VGCC protein level occurs in parallel with the changes observed in other presynaptic molecules, for instance, the nPKCε isoform and the exocytotic modulatory protein Munc18-1. Thus, a relevant differential transition affects the channel proteins around the crucial period (P5–P9) for synapse elimination.

### VGCC in Developmental Synapse Elimination

**Fig. 3:**
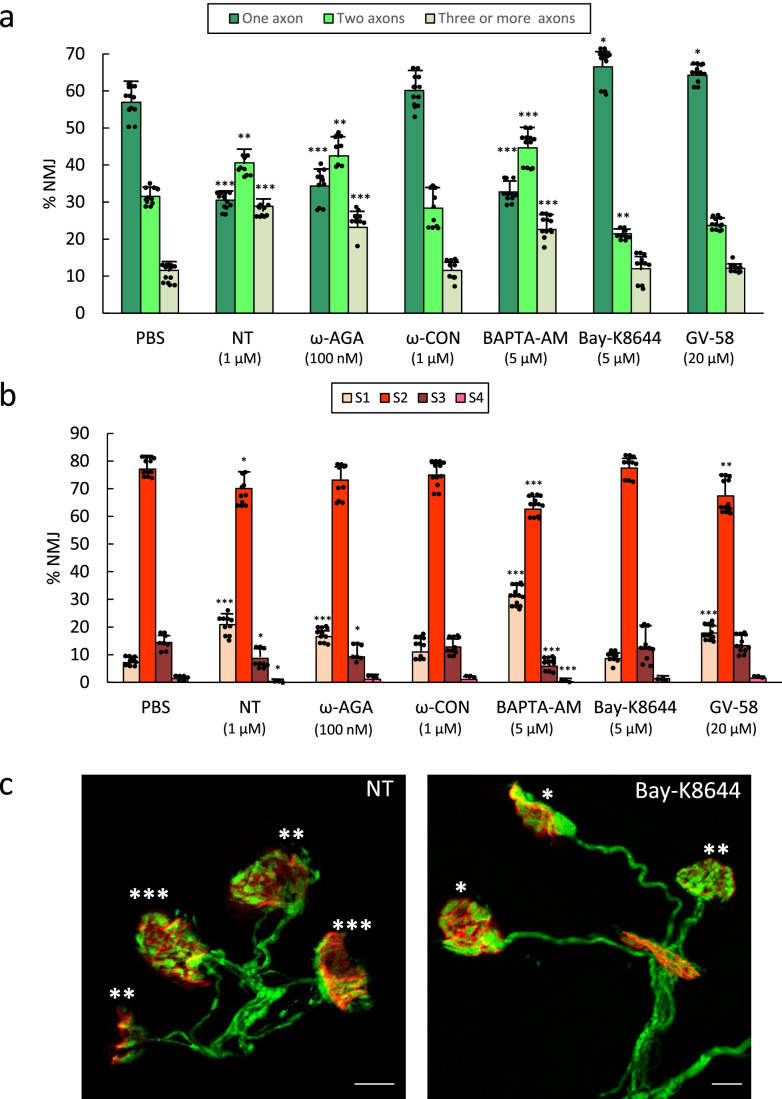
In **(a)** we show the percentage of singly- and polyinnervated NMJ after 4 applications over the LAL surface (one application every day between P5–P8 (observation at P9) of one of the following VGCC inhibitor substances: nitrendipine (NT 1 μM, an L-type channel blocker), ω-conotoxin-GVIA (ω-CON 1 μM, N-type channel blocker), and ω-agatoxin-IVA (ω-AGA 100 nM, P/Q-type blocker). Also, the L activator Bay-K8644 (5 μM), the P/Q- and N-type activator GV-58 (20 μM), and the intracellular calcium chelator BAPTA-AM (5 μM). The histogram in (**b**) shows the percentage of S1-S4 clusters in the untreated control mice (PBS) and after the 4 applications of the aforesaid substances. Data were presented as percentages of NMJ ± SD. Fisher’s test: * *p* < 0.05, ** *p* < 0.01, *** *p* < 0.005. The confocal images in **(c)** show examples of representative NMJ areas with singly, dually, and innervated by three or more axons (the corresponding number of asterisks) from YFP muscles. At the left, the L-type channel blocker nitrendipine (NT) delays axon loss because many multi-innervated NMJs persist. By the contrary, at the right, the L activator Bay-K8644 increases the number of monoinnervated junctions. The bar indicates 10 μm

We performed subcutaneous injections over the *Levator auris* *longus* (LAL) mouse muscle of selective blockers and activators of the VGCC. Figure 3a shows the percentage of singly, dually, and innervated by three or more axons NMJs in the untreated control Thy1-YFP-16 expressing mice (only PBS) and after 4 applications (one application every day between P5-P8; observation at P9) of one of the following VGCC inhibitor substances: nitrendipine (NT 1 μM, an L-type channel blocker), ω-agatoxin-IVA (ω-AGA 100 nM, a P/Q-type blocker) and ω-conotoxin-GVIA (ω-CON 1 μM, N-type channel blocker). The data show that the L channel block with NT and the P/Q channel block with ω-AGA results in an important delay of the axon loss because of the persistence of many polyinnervated synapses and thus a percentage of monoinnervated junctions around half of the value expected at P9. However, the block of the N channel with ω-CON does not affect the normal rate of axonal loss.

We used also VGCC activators. Bay-K8644 is an L-type Ca^2+^-channel activator that increases the entry of Ca^2+^ into cells by opening the channel for longer periods [46, 47]. Activation of the L channel with 5 μM Bay-K8644 produces the contrary effect of the L block because of the small but significant increase of monoinnervated NMJ and the reduction of the dual junctions.

GV-58 was shown to be more potent on N- and P/Q Ca^2+^ channels with an *EC*_50_ = 6.8 and 9.9 μM, respectively, over L-type calcium channels (*EC*_50_ > 100 μM) (Tarr et al., 2012). GV-58 slows the closing of the VGCCs, resulting in a large increase in total Ca^2+^ entry during motor nerve action potential activity [48]. The activation of the P/Q- and N-type VGCCs with GV-58 (20 μM) results also in a moderate though a significant increase of the monoinnervated synapses. This effect was accompanied by a tendency to decrease doubly-innervated synapses (− 25%). Thus, the exogenous stimulation with channel activators reveals that VGCCs (especially the L channel) have the potential to promote postnatal axonal disconnection, and this function is clearly observed because of the tonic delay in axon loss on L and also P/Q block.

In relation with the postsynaptic site, the histogram in Fig. 3b shows the percentage of S1–S4 clusters of the control mice (PBS) and after 4 applications (between P5-P8) of the VGCC blockers and activators. Similarly, as the effect of the channel blockers on axonal elimination, we observed here that both the L and the P/Q blockades result in a significant persistence of the most immature S1 nAChRs clusters along with a decrease of the S2 and/or S3 clusters, thus a moderate delay in maturation as compared with the values expected at P9. On the other hand, the block of the N channel with ω-CON does not affect the normal postsynaptic differentiation, as expected due to the lack of effect on axon loss. Activation of the L channel with Bay-K8644 does not induce any change in the normal percentages of S1–S4 postsynaptic clusters suggesting that the optimal coupling of this channel cannot be further stimulated. Interestingly, the activation of P/Q- and N-type VGCCs with GV-58 results in a significant delay in the postsynaptic maturation, causing the persistence of S1 clusters and diminution of S2 ones. Because the block and activation of the P/Q VGCC do not presumably result in the same effect on the nAChRs maturation, we think that the GV-58 effect on the postsynaptic site may be attributed to the activation of the N channel in this site.

Confocal microscopy examples (Fig. 3c) of the effect of NT and Bay-K8644 show differences in the intramuscular innervation. The L-type channel blocker delays axon loss, and many multiinnervated NMJs persist while the L activator Bay-K8644, accelerates maturation and increases the number of monoinnervated junctions.

To evaluate the involvement of calcium ions inflow in synapse elimination, we studied the intracellular calcium sequestration in BAPTA-AM exposed LAL muscles. The histograms in Fig. 3 show a great delay in both axon loss (comparable to the block of L and P/Q channels) and postsynaptic maturation (persistence of many S1 clusters), respectively, showing the relevance of the calcium channels and calcium ions entry in NMJ maturation. Table 1a shows that there is no significant difference between the effects of L and P/Q channel block themselves and between the block of any of these channels and the intracellular calcium sequestration effect over axonal elimination. However, at the postsynaptic site (Table 1b), though no significant difference exists between the effects of L and P/Q block themselves, the effect of BAPTA-AM is higher than the separate effect of Nitrendipine and ω-AGA suggesting the simultaneous involvement of both channels. As previously stated, activation of the P/Q- and N-type VGCCs with GV-58 results in the persistence of immature S1 clusters that does not differ from the individual block of the L and P/Q VGCC (Table 1b). This effect may be attributed to the activation of the N channel that therefore seems to play a role in postsynaptic maturation.

**Table 1:**
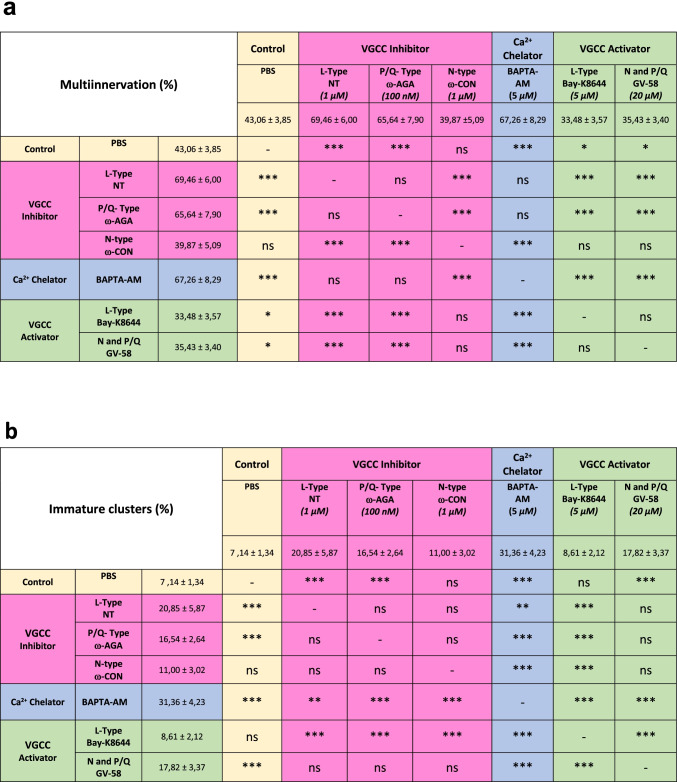
Comparison of the values of multi-innervation (**a**) and immature postsynaptic clusters (**b**) between the different VGCC and [Ca^2+^]_i_ modulators. Data are repeated both, at the right and at the back of the table, to allow for multiple comparison. (-): no comparison, (coincidence of the same value)

### Involvement of the Muscle Cell’s Contractile Activity

**Fig. 4:**
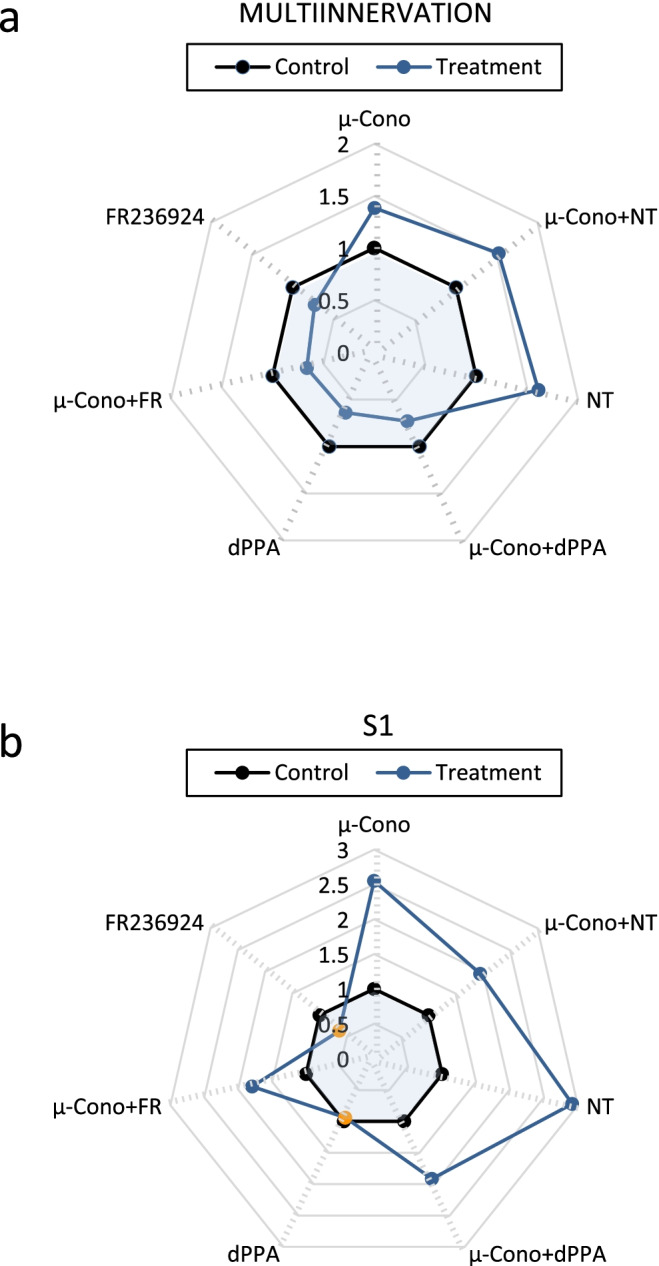
Diagrams are graphic representations that collectively show the pattern of action of the different agents and combinations of agents used. **a** Multi-innervation values. **b** Postsynaptic cluster (S1) values. The line between the blue and white areas means the ratio 1 (experimental value/control value) or “no effect.” Orange filled circles mean that the experimental value is not significantly different from the control value (*p* < 0.05). and filled circles that there is a significant difference (*p* > 0.05). SEMs are eliminated for clarity

We next analyzed the postsynaptic involvement in the axon loss regulation (Fig. 4). Using the same protocol as for the other substances, we incubated with (i) μ-conotoxin GIIIB (1.5 μM), (ii) μ-conotoxin GIIIB (1.5 μM) + nitrendipina (1 μM), and (iii) μ-conotoxin GIIIB (1.5 μM) + dPPA (0.2 μg/ml) or FR236924 (100 nM). These experiments would contribute to the discrimination of the effects produced by postsynaptic contractile activity. After the postsynaptic contraction block with μ-conotoxin GIIIB, it can be expected that only the presynaptic normal or only neurotransmission restricted events in the synaptic area or synaptic activity operate in axon loss. Thus, a postsynaptic contraction discrimination with the full synaptic transmission preserved can be observed (difference with the postsynaptic block with bungarotoxin or d-tubocurarine). These experiments show a delay in the synapse elimination (39% increase in multiinnervation vs control) similar in magnitude to that observed after the block of the TrkB pathway. At the postsynaptic level, the contraction block with μ-conotoxin GIIIB results also in a delay in nAChR cluster maturation with a significant persistence of the S1 immature clusters. Thus, a global delay in pre- and postsynaptic maturation was produced. Incubation with nitrendipine results in more retention of multi-innervation that those induced by μ-conotoxin GIIIB (61.3% increase in multi-innervation vs control, *P* < 0.05). When both substances are simultaneously applied, we observed an intermediate value (in fact, 52.4% increase in multi-innervation vs control while μ-conotoxin GIIIB only shows the aforesaid 38.6%). At the postsynaptic level, we observed some interference between these substances because the percentage of S1 clusters was less than the observed with the individual substances.

When μ-conotoxin GIIIB is applied simultaneously with dPPA or FR236924, it can be expected some prevention of the postsynaptic block effect of the μ-conotoxin GIIIB because the simultaneous stimulation of the strictly presynaptic PKC isoforms (isoforms PKCβI and PKCε that when activated promote elimination and if inhibited axon loss is delayed). We observed with the use of both PKC-stimulatory substances a full prevention of the μ-conotoxin GIIIB effect with a very relevant acceleration of the axon loss that approaches to the values obtained after treatment with the PKC activators only. At the postsynaptic level, both PKC-stimulatory substances (that no produce any maturation change in the morphology of the nAChR cluster when applied individually) slightly modify the μ-conotoxin GIIIB effect (a relevant delay of maturation) and produce some reduction of the most immature S1 clusters though without attaining the normal control value.

In summary, the block of the muscle cell’s contractile activity results in a delay in axon loss. The simultaneous application of the PKC activators dPPA or FR236924 and μ-conotoxin GIIIB fully prevents the postsynaptic contraction block effect on axon loss indicating the reliability of a presynaptic modulation of the presynaptic PKC’s independent of the postsynaptic activity or in parallel with it. The maturation of the postsynaptic receptor clusters clearly depends on the muscle cell contractile activity.

### Comparison Between VGCC Block and Stimulation with PKA and PKC Activity Modulation

In previous studies, we found that during the first postnatal days (P5–P9), PKA and PKC have opposed effects in delaying and favoring, respectively, synapse maturation [3, 35, 45, 49]. Here, we evaluate the strength of the hypothesis of the close relation between the effect of these serine/threonine kinases and VGCCs for developmental axonal competition and loss. We repeated some experiments of PKA and PKC stimulation or inhibition of the previously cited paper Garcia et al. [49] and compared them with those obtained after VGCC stimulation or inhibition. PKA and PKC modulation of VDCCs has been determined, and phosphorylation sites of functional relevance are known for the three L-, P/Q-, and N-type VDCCs [50–52]. We focused on nerve terminals elimination and thus on the percentage of multi-innervated junctions after the exposition of the LAL muscle to the different substances.

When considering the involvement of PKA in synapse elimination, it seems (Table 2) that the tonic coupling of PKA is weak. This is because its full inhibition (H89) just causes a small effect on axon loss and RI- or RII-preferential blockers (Rp8-Br or Rp-cAMPs that prevent cAMP binding to the regulatory subunits blocking their dissociation from the catalytic subunits and their action) do not produce any significant effect. However, it seems that the potential of PKA to influence elimination is strong because activation (Sp8Br) induces a great delay in elimination. Contrarily, PKC has a relevant tonic involvement manifested on inhibition (for instance, CaC and Che cause an important reduction of monoinnervation). A similar effect on axon loss is produced by specific block of the presynaptic cPKCβI and nPKCε isoforms (with βIV_5–3_ and εV_1–2_ peptides, respectively). Interestingly, the PKC involvement on axon loss can be moderately increased by general stimulation (Bry-1 or PMA) or by the specific cPKCβI and nPKCε stimulation (dPPA and FR236924). Thus, activation versus inhibition of both PKA and PKC result in opposed effects on axon loss. However, the strongest effects are produced with PKA stimulation and PKC inhibition suggesting that axonal retraction would occur mainly in a context of low PKA/PKC ratio.

**Table 2:**
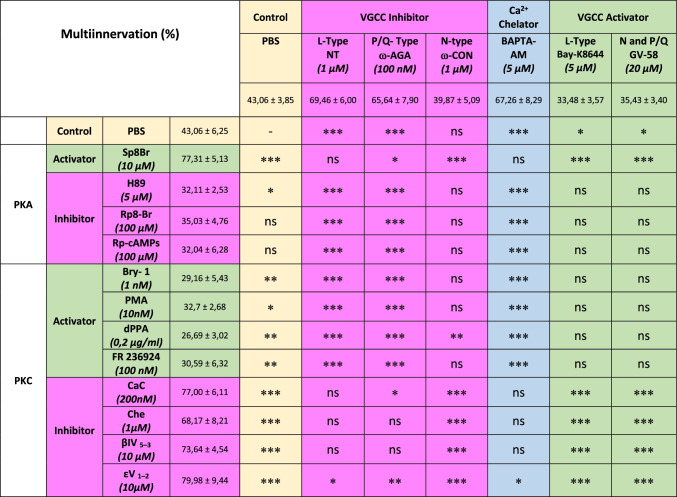
Comparison between VGCC and [Ca^2+^]_i_ modulation with PKA and PKC activity modulation. Comparison of the values of multi-innervation (percentage) observed after VGCC block and activation with the values after the block and activation of PKC and PKA. (-): no comparison (coincidence of the same value)

In analyzing the relation between PKA, PKC, and VGCC, Table 2 shows that the block of the L-type VGCC with NT results in the same delay in axonal loss to the PKA stimulation with Sp8Br and the block of PKC with CaC, Che, or the cPKCβI blocker βIV_5–3_. Interestingly however, the nPKCε blocker εV_1–2_ produces a significantly greater delay in the axon loss than the L channel block. The activation of the L channel with Bay-K8644 results in the opposed effect that PKA stimulation and PKC inhibition whereas intracellular calcium sequestration with BAPTA-AM results in the same effect than L channel (see Table 1) and PKC block and also PKA stimulation. However, also in this case, the nPKCε blocker εV_1–2_ produces a significantly greater delay in the axon loss than BAPTA-AM. The greater effect produced by the nPKCε block as compared with the L channel block and intracellular calcium sequestration suggests a calcium-independent effect contributing to axonal loss. On the other hand, the block of the P/Q-type channel with ω-AGA results in a similar delay in axonal loss as the PKA stimulation or PKC inhibition similarly as with the block of the L channel. However, the statistical analysis shows, in this case, that several comparisons are significantly different because the P/Q block effect is smaller than, for instance, PKA activation with Sp8Br or nPKCε isoform block with the peptide εV_1–2_ or even general PKC block with CaC. The activation of the P/Q and N channels with GV-58 results in the opposed effect of PKA stimulation and PKC inhibition. The intracellular calcium sequestration with BAPTA-AM results in the same effect than P/Q channel block (see Table 1), PKA stimulation, and PKC block except for the nPKCε blocker εV_1–2_ which also produces a greater delay in the axon loss than BAPTA-AM.

In summary, the block of L- or P/Q VGCC or [Ca^2+^]_i_ sequestration results in a similar delay of axonal loss as that of the cPKCβI isoform block or PKA activation. On the other hand, data suggests an additional contribution of the calcium-independent nPKCε isoform.

## Discussion

We found that both L- and P/Q-type VGCCs (but not the N-type) are equally involved in postnatal axonal competition and synapse elimination. Judging by the effect of the specific block and activation of the channels, and the effect of intracellular Ca^2+^ chelators, their normal function can favor supernumerary axonal loss by increasing [Ca^2+^]_i_. The block of these VGCC or [Ca^2+^]_i_ sequestration results in the same delay of axonal loss to the cPKCβI isoform block or PKA activation. However, nPKCε block causes a greater delay, suggesting the involvement in this case of an additional calcium-independent mechanism. The involvement of the VGCC in the postsynaptic maturation seems more complex. In addition to the agrin mechanism, activation of nAChRs through neuromuscular transmission can be sufficient to induce receptors aggregation with the involvement of muscle L-type channels [53]. Also, developing muscle cells are intrinsically “pre-patterned” in the center of the muscle fibers for motor nerve innervation and NMJ formation, and a functional skeletal muscle L-type VGCC (and also sarcoplasmic reticulum calcium release) was required [54–56]. However, some contribution of the N-type VGCC cannot be discarded and merits further investigation.

Developmental synapse elimination depends mainly on the activity-dependent nerve terminal competition based on differences of transmitter release from the competing axons [5, 57–59]. Thus, a relation exists between transmitter release and nerve endings retraction or stabilization. Several types of VGCC have been identified, but the P/Q type (Ca_V_2.1) is the main channel involved in nerve-evoked transmitter release at many synapses including the adult NMJ. However, in several physiological (including development), pathological, or experimental conditions, other channel types (N-type Ca_V_2.2 and L-type Ca_V_1.1) can be present or unmasked. Calcium channels may be recruited to neurotransmission in different functional demands [60]. Several α1 and β VGCC subunits were present at the adult NMJ suggesting some redundancy for the transmitter release-mediating function, and the compensatory expression between them [61–63] suggests the ability to substitute the P/Q channel. For instance, spontaneous release was dependent only on P/Q-type VGCC in normal NMJs. However, when neurotransmitter release was potentiated by the presence of the K^+^ channel blocker 4-aminopyridine (4-AP) [64], under conditions of intense nerve terminal depolarization or during high-frequency bursts of NMJ activity, L-type channels may be recruited to facilitate transmitter release [65]. In pathological conditions as the Lambert-Eaton Myasthenic syndrome, an autoimmune attack on P/Q channels is followed by unmasking of an L-type current [64, 66].

During NMJ synaptogenesis, there is a normal progressive switching from N- to P/Q-type VGCC-mediated transmitter release [67]. Thus, during mammalian NMJ formation (and regeneration) evoked transmitter release was strongly reduced by a P/Q-type VGCC blocker. It seems that the P/Q-type VGCCs were more efficiently coupled to transmitter release than were N-type at the neonatal neuromuscular junction [68]. Interestingly enough various L-type blockers, both dihydropyridine and nondihydropyridine antagonists, increased evoked (but not spontaneous) release in a dose-dependent manner at newly formed NMJs. This presynaptic potentiation disappeared as NMJs matured. Thus, L-type VGCC plays also a modulatory role in evoked transmitter release by activating a mechanism linked to PTX-sensitive G-proteins that reduce transmitter release during synapse maturation [69].

However, during neuromuscular synaptogenesis, there are several nerve terminals in competition showing, at a given time, different levels of maturation or involution, and the specific involvement of each VGCC in different endings is not well enough known. In relation with the aforesaid role of the L channel in reducing transmitter release, we observed previously that the weak nerve terminal in dually innervated NMJs (the ending that evokes the small synaptic potentials at postnatal week one) was potentiated by partially reducing calcium entry by any VGCC [P/Q-, N- (in this case only transitorily during the first minutes of the block), or L-type VGCC-specific block] or 500 μM magnesium ions in the bath, M_1_-type selective mAChR block with pirenzepine, or PKC block with CaC or Che [25, 27, 70–72]. This effect does not occur in the strongest nerve terminal and neither in the only one ending in most mature junctions some time later at 2-week-old animals. Moreover, reducing calcium entry or blocking PKC or mAChRs results in unmasking functionally silent nerve endings that transitorily now recover transmitter release [27, 72]. The PKA-linked M_2_ subtype is also present in the weakest endings, but it is related only to P/Q and N channels to potentiate release. In fact, L channel is coupled to M_1_ mAChRs only in the weak and strong endings in dual synapses but not in the more mature solitary endings. In the strongest and mature endings, the coupling of M_1_ to PKC activity results in ACh release potentiation using Ca^2+^ inflow only through the P/Q-channel [25, 72–74]. Therefore, functional L-type channels transitorily present in the weak endings, intervene in differential transmitter release, and may contribute to the competitive interactions between axonal endings. It can be speculated that the high-calcium entry through the several operative channels present in some nerve endings during development (including the transitory L channel) results in the final loss of some nerve terminals. Our present results strongly support this interpretation. Thus, L and P/Q channel-mediated increased calcium inflow contributes to both transmitter release reduction from certain axons and the final nerve terminal loss and this coincidence argues in favor of a unitary mechanism.

It’s known that PQ-type channels are the primary channels responsible for neurotransmission at the NMJ. Our results showed similar levels of innervation when NT and W-Aga were used. Even if both channels contributed equally to presynaptic calcium signals at this stage of development, blocking either channel would be unlikely to produce equal effects on total calcium signal blocking with BAPTA. However, we favor the hypothesis that only a part of the calcium inflow is devoted to contribute to the axonal retraction and this aliquot, probably acting through a saturable mechanism, can be indistinctly carried by the operative VGCC present in the endings in process of elimination. Because of this consideration, we think that the effects of L- and PQ-type channel block (or activation) may not be additive.

However, there are some complementary interpretations from our results. The drug modulation of the L-type channel’s activity we performed could affect the function of the muscle CaV1.1 channel. This L-type Ca^2+^ channel in skeletal muscle, functions primarily as a voltage sensor that couples depolarization of the transverse tubules to ryanodine receptor opening and release of Ca^2+^ from the sarcoplasmic reticulum promoting contraction. Dihydropyridine actions on the muscle CaV1.1 could contribute to synapse elimination by affecting both directly a change in the postsynaptic activity and postsynaptic specializations and the nerve terminals via retrograde signals. This possibility can be not disconsidered and merits further analysis because, in fact, modulation of the BDNF-TrkB retrograde signaling affects axonal elimination [5, 75]. However, the changes in the rate of axonal elimination can be produced by acting directly on presynaptic molecular targets as the P/Q VGCC and also presynaptic muscarinic and purinergic autoreceptors. It is known since many years ago that changes in developmental synapse elimination are produced after blocking or reducing muscle activity. In almost all cases, reducing activity results in an extended period of synapse elimination [76]. Classical experiments were made by Thompson [77], who paralyzed the soleus muscle by TTX and observed that the synaptic elimination was prevented. Brown et al. [78] and Duxson [79] using α-bungarotoxin to inactivate the muscle found an increased number of synaptic terminals on muscle fibers in newborn rat muscle. Later, Ding et al. [80] described the effects of curarization in chicken embryos where all the normal motoneuronal cell death was essentially prevented. Also, Callaway and Van Essen [81] studied the treatment by α-bungarotoxin from postnatal day 6 at 11 in rabbit soleus muscle and observed that the elimination of polyneuronal innervation was significantly reduced.

A postsynaptic discrimination with the full synaptic transmission preserved can be observed after the contraction block with μ-conotoxin GIIIB. Here, the results show that, as expected, the block of the muscle cell’s contractile activity results in a delay of synapse maturation (both supernumerary axon loss rate and nAChRs maturation). We think that a part of this effect at the presynaptic site may be mediated by a retrograde influence (via BDNF-TrkB) on the presynaptic CaV1.3, which, when directly inhibited by nitrendipine results, however, in a greater effect in delaying axon loss. Experiments in the adult show an increase in the mBDNF production when synaptic activity results in muscle cell contraction [82]. A contrary effect to that of the μ-conotoxin GIIIB is produced by presynaptic PKC isoforms which are directly activated by dPPA or FR236924 because of strongly accelerated axon loss. The simultaneous application of these substances and μ-conotoxin GIIIB completely prevents the postsynaptic contraction block effect on axon loss indicating the reliability of the presynaptic modulation of the PKC’s by muscarinic, purinergic, or other synaptic mechanisms to affect synapse elimination independent of the postsynaptic contractile activity or in parallel with it. The maturation of the postsynaptic receptor clusters clearly depends on the muscle cell contractile activity. However, some presynaptic-mediated influence in the postsynaptic maturation is revealed because PKC stimulation in the nerve terminal reduces significatively (though not completely) the effect of the μ-conotoxin GIIIB on the postsynaptic cluster maturation.

What could be the mechanism activated by the high [Ca^2+^]_i_? It is plausible that a calcium-activated neutral protease (CANP) present in nerve endings could contribute [83]. A greater increase of Ca^2+^ concentration in smaller terminals would be expected, because of their surface-to-volume ratio and the collegiate activity of several VGCC. Interestingly, it seems that, in some cells, L channels couple to increase spontaneous and evoked quantal transmitter release when phosphorylated by PKC and PKA and provided that serine/threonine protein phosphatases are blocked (okadaic acid) and low intracellular calcium (BAPTA-AM). When only L-type channels were available, quantal content increased when [Ca^2+^]_o_ increased from 0.5 to 1 mM, but decreased significantly at 2 mM [60]. This may be due to the activation of the phosphatases by calcium leading to the inactivation of relevant molecular targets involved in neurotransmission. It is known that cross talk between protein kinases and phosphatases regulates synaptic strength in the mammalian brain [84]. Changes in the synaptic activity of neurons promoted the redistribution of protein phosphatase 1 (PP1) associated with the actin-rich cytoskeletal structures near the plasmalemma [85] to allow effective dephosphorylation of PP1 substrates. Moreover, synaptic elimination seems to be driven by a reorganization of the F-actin cytoskeleton [86] and disassembly of the microtubules [87]. Thus, it may be a specific coupling of the L-type VGCC in the weakest nerve terminals that contribute to a high increase in calcium entry (mediated in fact by the three considered channels that are operative at this time) resulting in the downregulation of the transmitter release in these endings and also—with regard to L and P/Q VGCC—promoting terminal destabilization by protein phosphatases activation.

Adenosine receptors (AR: A_1_ and A_2A_), presynaptic muscarinic ACh autoreceptors (mAChR: M_1_, M_2_, and M_4_ types), and the tropomyosin-related kinase B neurotrophin receptor (TrkB), among other receptors, support competitive signaling between motor axons. In previous studies, we investigated the synergistic and antagonistic relations between these receptors affecting synapse elimination [5, 88, 89]. The receptors M_1_, A_1_, and TrkB operate mainly through PKC whereas M_2_, M_4_, and A_2A_ are coupled to PKA [15–17, 19, 20, 90–92]. At P9, all these receptors are tonically coupled to promote axonal removal through PKC stimulation or PKA inhibition [4, 14, 21]. A change in the PKA/PKC activity ratio is the main parameter that seems to change after all the direct and crossed inhibitions of the mAChR, AR, and TrkB that had been checked. We also reported that multi-innervation could be stabilized by delaying axonal elimination when PKA-I and II were activated (see Table 2) in P5–P9 neonatal mice. Contrarily, PKC activity, through cPKCβI and nPKCε isoforms action, promotes axonal loss [24]. Moreover, a similar level of PKC potentiation and PKA inhibition is required during developmental synaptic elimination [49] and no significant differences exist between the effects of PKA activators and PKC inhibitors or PKA inhibitors and PKC activators on the developmental axon loss rate, which indicates the complementarity of the kinases [49]. Changes in the phosphorylation of PKA and PKC targets involved in transmitter release and nerve terminal stability could realize the final molecular mechanism of synapse loss. PKC phosphorylation and activation of VGCC would allow the high calcium inflow that seems necessary for axon loss. Coincidentally, calcium inflow would activate classic PKCs such as cPKCβI to reinforce their role in axonal elimination.

Here, we performed a detailed statistical analysis of the comparison between the serine kinases results with those of the VGCC stimulation or inhibition. The results show the same tendency to delay maturation with the block of L and P/Q channels than with the block of PKC (cPKCβI and nPKCε isoforms) and activation of PKA. The greater effect produced by the nPKCε block as compared with the L and P/Q channel block and intracellular calcium sequestration suggests the possible existence of a calcium or VGCC-independent effect contributing to axonal loss. These results encourage new experiments to explore the mechanism that links synaptic activity and membrane receptor signaling, the downstream serine kinases, and calcium channels with synapse elimination. Thus, the hypothesis that calcium channels activity, linked to PKC and PKA activity, is a key component of the synapse elimination events is strengthened and merits further study.

## Conclusions

We investigated the involvement of the P/Q-, N-, and L-subtypes of the VGCC in synapse elimination during neuromuscular junction development. All three proteins increase between P5 and P7. Observation at P30 reveals that P/Q channel levels continue increasing, α1D-L channel protein stabilizes at the P7 value, and N channel protein falls back to the low value observed at P5. Thus, a relevant differential transition affects the channel proteins around the crucial period (P5–P9) for synapse elimination.

Both L and P/Q-type, but not the N-type channel, tonically promotes the postnatal synapse maturation because their block at the half-time period of axonal elimination strongly prolongs both multi-innervation and delays postsynaptic nAChR cluster maturation. The exogenous stimulation with channel activators results in the contrary effect on axon loss, though some involvement of the N channel at the postsynaptic site may be unmasked.

There is no significant difference between the effects of L channel block, P/Q channel block, and intracellular calcium sequestration in favoring a delay in axonal elimination. However, at the postsynaptic site, though no significant difference exists between the effects of L and P/Q block themselves, the effect of BAPTA-AM is higher than the separate effect of nitrendipine and ω-AGA suggesting the simultaneous involvement of both channels.

We confirmed previous results showing that PKA activity seems to stabilize multi-inervation. Contrarily, PKC activity promotes axonal loss (through cPKCβI and nPKCε isoform action). We evaluated the hypothesis of the close link between the serine/threonine kinases PKA and PKC and VGCC for developmental synapse elimination and found that the result after the block of the L-channel (and also after intracellular calcium sequestration) is not different from the block of cPKCβI and from stimulation of PKA with an important delay in maturation in all cases. Interestingly, the block of the nPKCε results in an even greater delay in the axon loss than the L or P/Q channels block or calcium sequestration, suggesting a PKC-mediated VGCC-independent component contributing to axonal loss. Similar to the block of the L channel, the block of the P/Q-type channel results in a delay in axonal loss similar to that observed after cPKCβI inhibition; however, the effect of P/Q block is smaller than PKA activation (in addition to the nPKCε block). Thus, activation of PKA produces a significantly greater effect than P/Q block but not than L block.

Interestingly, the block of the muscle cell’s contractile activity with μ-conotoxin GIIIB also results in a delay in axon loss, and thus, a retrograde influence from the postsynaptic site may contribute to the synapse elimination. In fact, the drug modulation of the L-type channel’s activity we performed could affect the function of the muscle CaV1.1 channel and contribute also to the observed changes. The simultaneous application of the PKC activators dPPA or FR236924 and μ-conotoxin GIIIB fully prevents the postsynaptic contraction block effect on axon loss indicating the reliability of a direct presynaptic modulation of the presynaptic PKC’s independent of the postsynaptic activity or in parallel with it. The fact of the observed modulation of axonal loss by acting directly in presynaptic targets suggests that the involvement of the postsynaptic retrograde influence would be not necessary. Nevertheless, there exists the possibility that the above-cited presynaptic targets themselves may be modulated in part by a retrograde control, and this argues in favor of a complex regulation through pre- and postsynaptic activity of the mediators of the synapse elimination.

**Fig. 5:**
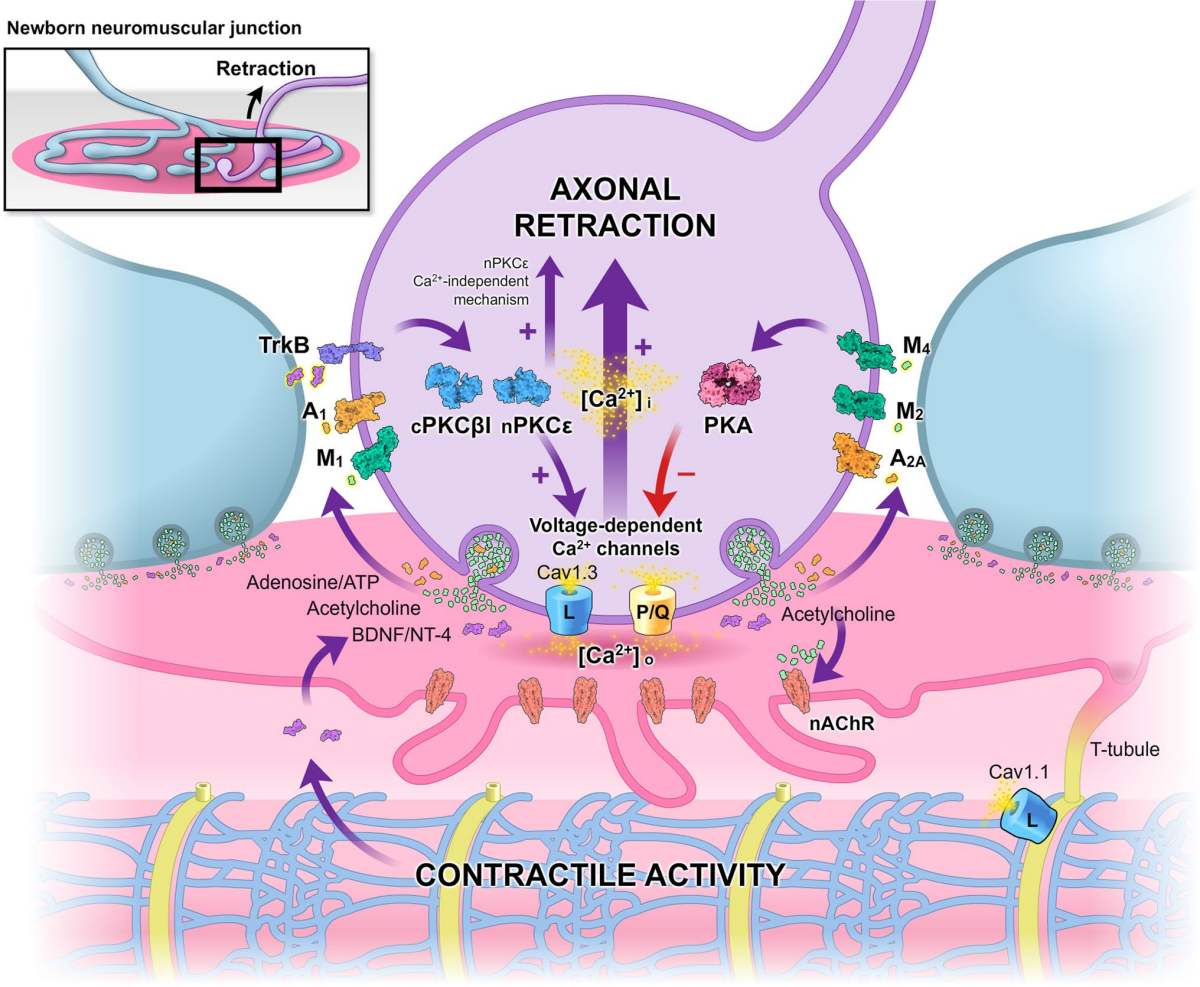
Graphic representation of the results. The activity-dependent signaling between the nerve terminals that are in competition through several metabotropic receptors can result in the modulation of the downstream effector kinases, specifically cPKCβI, nPKCε, and PKA. Changes in kinases activity can allow the coordinate phosphorylation of the L-type CaV1.3 and P/Q-type VGCC. The high calcium entry through these operative channels present in immature nerve endings can result in their final loss. Also, muscle CaV1.1 and contractile activity can contribute to the synapse elimination. A component of this mechanism may be mediated by a retrograde influence from the postsynaptic site, via the BDNF-TrkB pathway, on the presynaptic calcium channels

In summary, a relevant actor in developmental synapse elimination is [Ca^2+^]_i_ elevation in some nerve terminals through L- and P/Q-type VGCC (Fig. 5). The high calcium entry through these operative channels that are present in immature nerve endings (including the transitory L channel) results in their final loss. Thus, a [Ca^2+^]_i_ increase contributes to both transmitter release reduction in certain axons and nerve terminal loss, and this coincidence argues in favor of a unitary mechanism. Channel activation would be modulated by cPKCβI and nPKCε activity and also PKA inhibition. The greater effect of nPKCε block on delaying axons elimination suggests the contribution of a calcium-independent mechanism in the normal maturation.

## Supplementary Information

Below is the link to the electronic supplementary material.

Supplementary file1 (PDF 365 KB)

## Data Availability

We think that our data are not appropriate for the available repository database in neuroscience.

## Abbreviations

ACh: Acetylcholine
AR: Adenosine receptors
A_1_: Adenosine receptor
A_2A_: Adenosine receptor
ω-Aga-IVA: ω-Agatoxin-IVA
BRY: Bryostatin-1
CANP: Calcium-activated neutral protease
CaC: Calphostin C
Che: Chelerythrine
ω-CON-GVIA: ω-Conotoxin-GVIA
DMSO: Dimethylsulfoxide
LAL: *Levator auris* *longus* Muscle
nAChR: Nicotinic acetylcholine receptor
M_1_: M_1_-type muscarinic acetylcholine receptor
M_2_: M_2_-type muscarinic acetylcholine receptor
M_4_: M_4_-type muscarinic acetylcholine receptor
Munc18-1: Mammalian uncoordinated-18–1
NMJ: Neuromuscular junction
PBS: Phosphate buffer saline
PKA: Protein kinase A
PKA-I: PKA isozymes type I
PKA-II: PKA isozymes type II
PKC: Protein kinase C
cPKCβI: conventional protein kinase C beta I
βIV_5__-3_: CPKCβI specific translocation inhibitor peptide
nPKCε: Novel protein kinase C epsilon εV_1-2_
nPKCε: Specific translocation inhibitor peptide
PMA: Phorbol 12-myristate 13-acetate
Rp: Adenosine- 3′,5′-cyclic monophosphorothioate Rp-isomer sodium salt
Rp8: 8-Bromoadenosine-3′,5′-cyclic monophosphorothioate, Rp-isomer sodium salt
S: Postsynaptic morphological stage
Thy1-YFP-16: Transgenic B6.Cg-Tg 16 Jrs/J mice
TRITC-α-BTX: tetramethylrhodamine α-bungarotoxin
TrkB: Tropomyosin-related kinase B receptor
VGCC: Voltage-gated calcium channels
