# Supplementary material for: Involvement of the Voltage-Gated Calcium Channels L- P/Q- and N-Types in Synapse Elimination During Neuromuscular Junction Development
Source: Mol Neurobiol. 2022 Apr 27;59(7):4044–64. doi: 10.1007/s12035-022-02818-2 (PMC9167222; doi:10.1007/s12035-022-02818-2)

Supplementary Figure 2a. Original Western blot and Sypro Ruby membranes. Please notice that we cut the membranes to incubate different proteins from one single membrane.

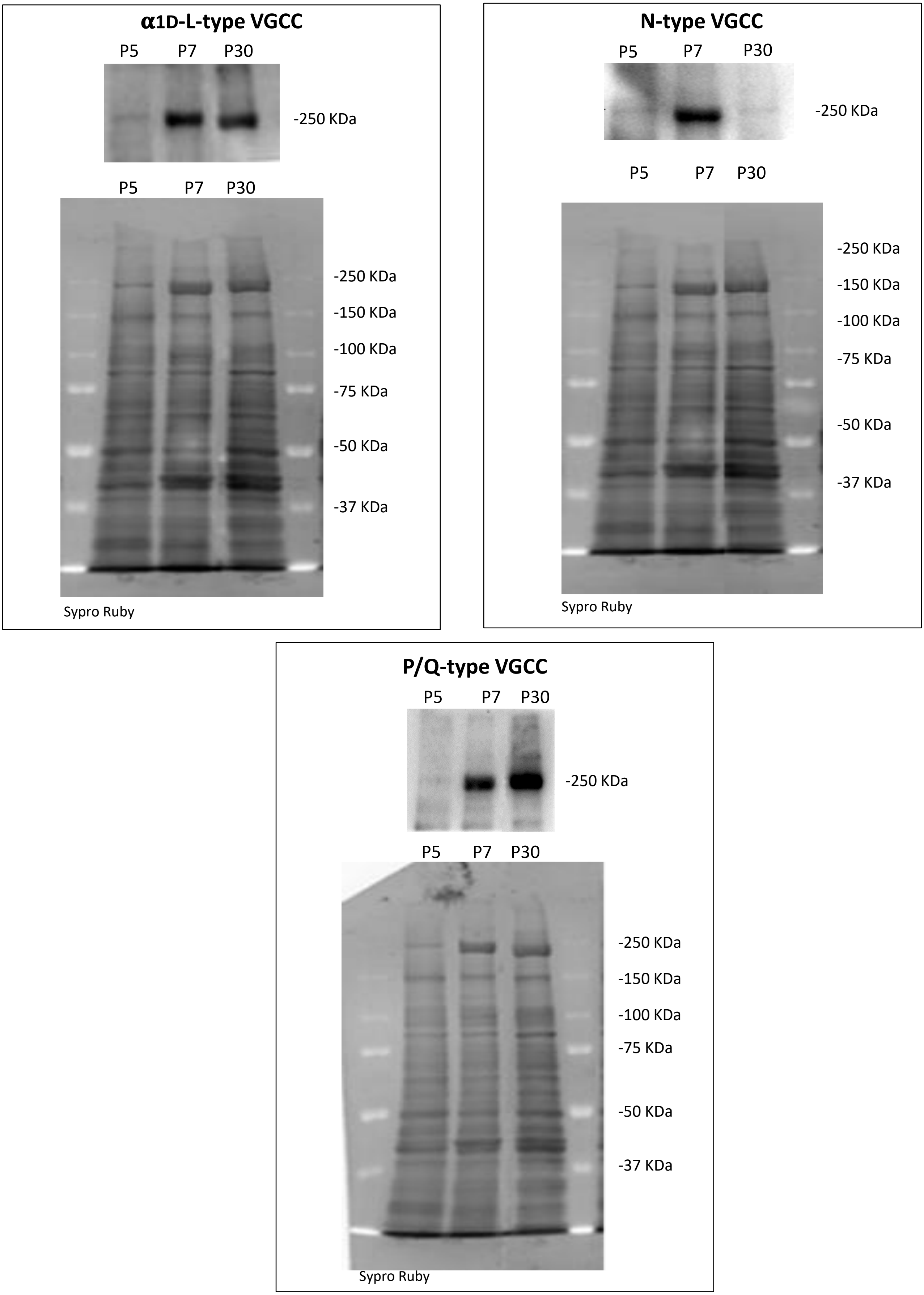

Supplementary Figure 2a. Original Western blot and Sypro Ruby membranes. Please notice that we cut the membranes to incubate different proteins from one single membrane.

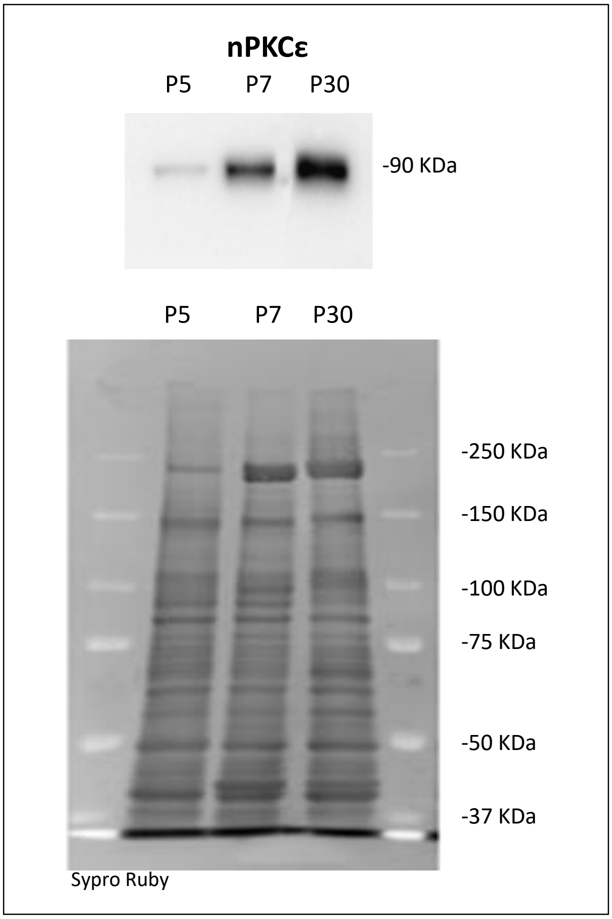

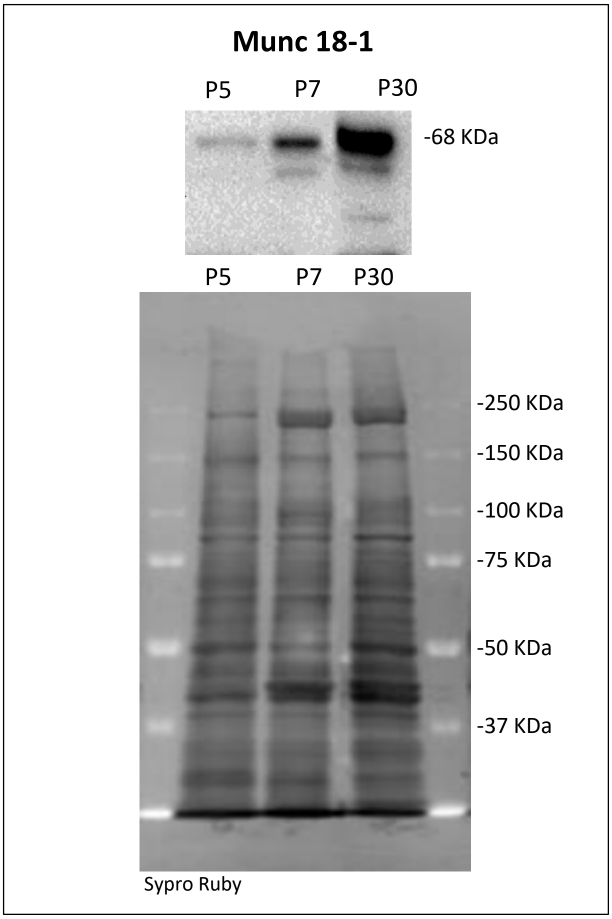

Supplementary Figure 2. Western Blot Controls. Examples of positive control using neonatal P5 and P30 adult brain, negative control without primary antibody (NC) and Sypro Ruby membranes. Please notice that we cut the membranes to incubate different proteins from one single membrane.

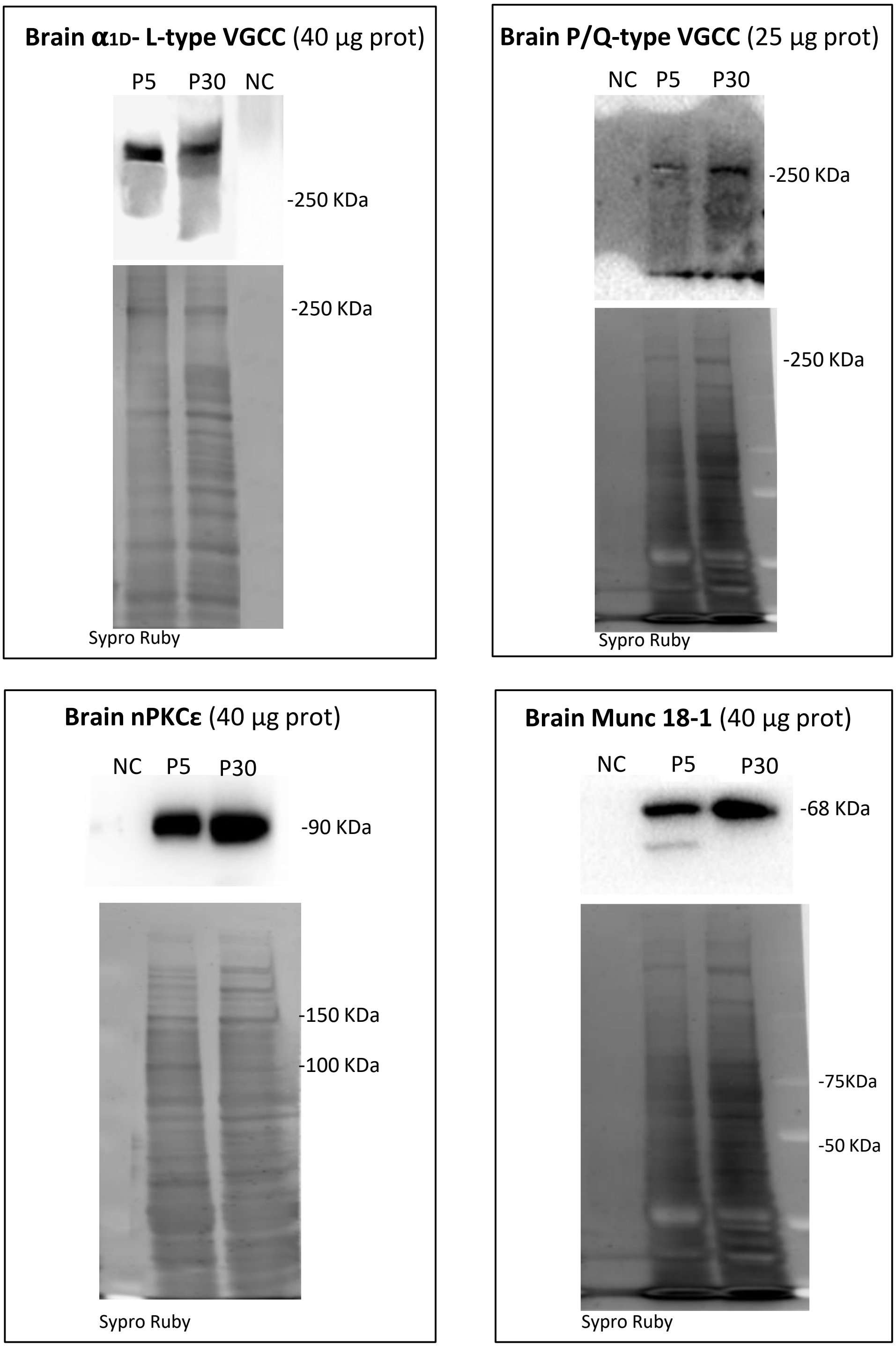

Supplementary Figure 2. Western Blot Controls. Examples of negative control using neonatal P5 and P30 skeletal muscle. Preincubation with the blocking peptide (BP, ratio between the antibody and the blocking peptide 1:1; examples from α_{1D} L-type VGCC/Cav1.3/CACNA1D blocking peptide (#BLP-CC005) and CACNA1A/Cav2.1 blocking peptide (#BLP-CC001)) in skeletal muscle tissue (P5 and P30) prevented immunolabeling. Please notice that we cut the membranes to incubate different proteins from one single membrane.

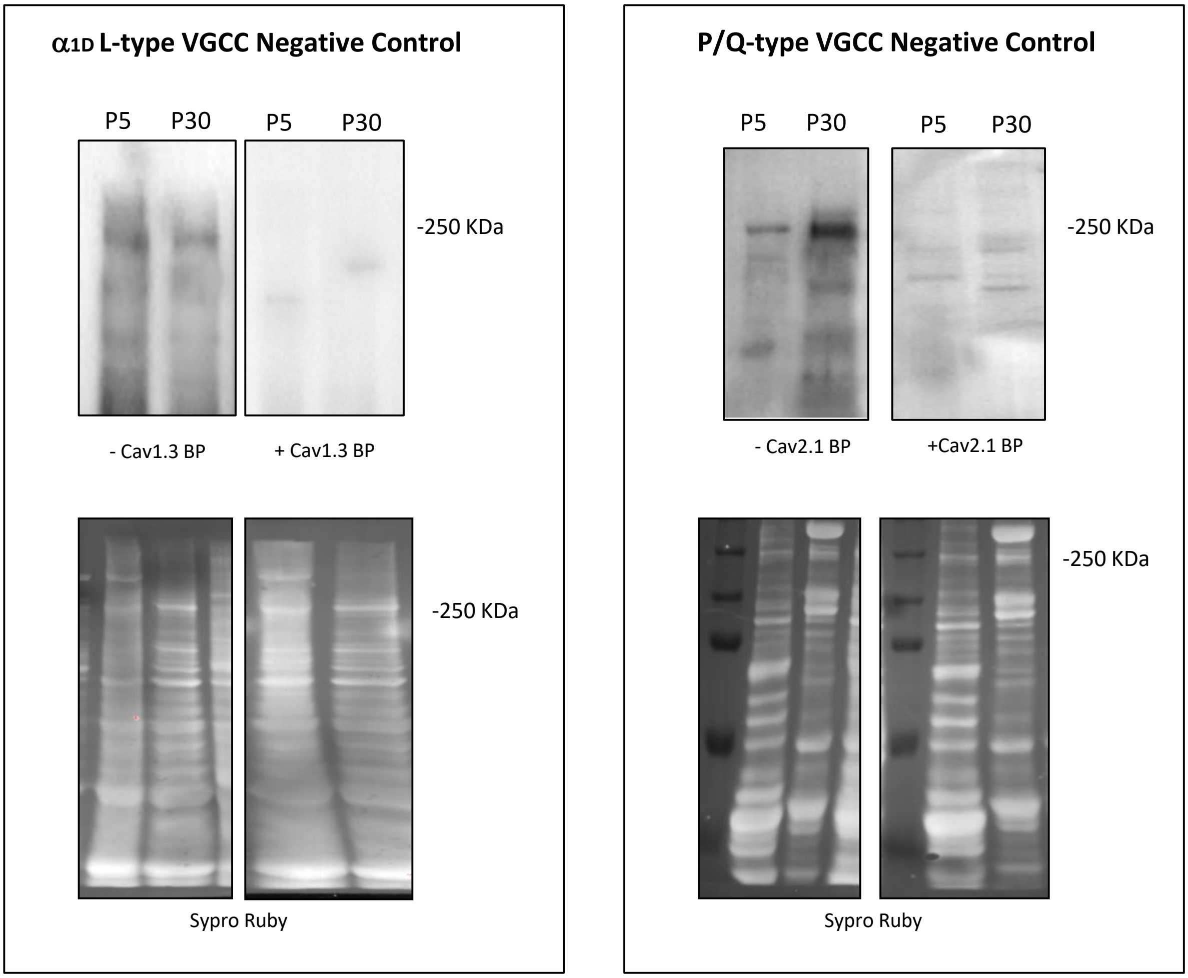

Supplement: Supplementary file 1 — Supplementary file1 (PDF 365 KB) [file 12035_2022_2818_MOESM1_ESM.pdf]
